# Horizontal gene transfer rate is not the primary determinant of observed antibiotic resistance frequencies in *Streptococcus pneumoniae*

**DOI:** 10.1126/sciadv.aaz6137

**Published:** 2020-05-20

**Authors:** Sonja Lehtinen, Claire Chewapreecha, John Lees, William P. Hanage, Marc Lipsitch, Nicholas J. Croucher, Stephen D. Bentley, Paul Turner, Christophe Fraser, Rafał J. Mostowy

**Affiliations:** 1 Big Data Institute, University of Oxford, Oxford, UK.; 2 Institute of Integrative Biology, Department of Environmental Systems Science, ETH Zurich, Zurich, Switzerland.; 3 Wellcome Sanger Institute, Hinxton, UK.; 4 Mahidol-Oxford Tropical Medicine Research Unit, Bangkok, Thailand.; 5 Bioinformatics and Systems Biology Program, School of Bioresource and Technology, King Mongkut’s University of Technology Thonburi, Bangkok, Thailand.; 6 Department of Infectious Disease Epidemiology, School of Public Health, Imperial College London, London, UK.; 7 Center for Communicable Disease Dynamics, Department of Epidemiology, Harvard T. H. Chan School of Public Health, Harvard University, Boston, MA, USA.; 8 Cambodia Oxford Medical Research Unit, Angkor Hospital for Children, Siem Reap, Cambodia.; 9 Centre for Tropical Medicine and Global Health, University of Oxford, Oxford, UK.; 10 Malopolska Centre of Biotechnology, Jagiellonian University, Krakow, Poland.

## Abstract

The extent to which evolution is constrained by the rate at which horizontal gene transfer (HGT) allows DNA to move between genetic lineages is an open question, which we address in the context of antibiotic resistance in *Streptococcus pneumoniae*. We analyze microbiological, genomic, and epidemiological data from the largest-to-date sequenced pneumococcal carriage study in 955 infants from a refugee camp on the Thailand-Myanmar border. Using a unified framework, we simultaneously test prior hypotheses on rates of HGT and a key evolutionary covariate (duration of carriage) as determinants of resistance frequencies. We conclude that in this setting, there is little evidence of HGT playing a major role in determining resistance frequencies. Instead, observed resistance frequencies are best explained as the outcome of selection acting on a pool of variants, irrespective of the rate at which resistance determinants move between genetic lineages.

## Teaser

Resistance frequencies are best explained as the outcome of selection, irrespective of the rate at which resistance is acquired.

## INTRODUCTION

Horizontal gene transfer (HGT), the nonvertical movement of genetic information between organisms, allows evolutionary innovation to spread onto new genetic backgrounds. However, predicting the extent to which the frequency of such transfer affects evolutionary outcomes (i.e., observed allele frequencies) remains a challenge in evolutionary biology. Beijerinck’s dictum “Everything is everywhere, but the environment selects” (*1*) pithily captures the hypothesis that population sizes are sufficiently large that evolution is essentially the deterministic outcome of competition between all possible variants. A competing hypothesis is that, in practice, competition is limited to the pool of available variants, and this pool is constrained by the rate at which genetic innovation arises, either through mutation or HGT. For this second hypothesis, lineages’ success therefore depends on the rate at which they acquire this innovation.

The evolution of antibiotic resistance is an important and illuminating area in which to address this question, because these two views of evolution lead to very different interpretations of observed patterns of resistance—particularly the heterogeneous distribution of resistance determinants among bacterial lineages. We will focus on HGT rather than mutation because phylogenetic studies suggest that this is the way clinically relevant resistance determinants are typically acquired (with a few exceptions—e.g., fluoroquinolone resistance typically arises through mutations) (*2*, *3*, *4*). The deterministic evolutionary ecology perspective (“everything is everywhere”) explains the distribution of resistance genes on lineages in terms of selection, that is, as the result of these genes conferring different fitness benefits on different lineages [e.g., Lehtinen *et al.* (*5*) and San Millan (*6*)]. Although lineages require HGT (or mutation) to acquire resistance determinants, it is assumed that these events occur often enough in the population so as not to limit the pool of available variants that selection can act on. Conversely, the “genetics as limiting factor” hypothesis posits that different lineages have different resistance levels because they are able to acquire resistance determinants at different rates. This view implies that antibiotic resistance is beneficial and will eventually spread ubiquitously but that lineages with higher rates of horizontal gene gain have been able to acquire these genes faster (*7*). Previously, these potential predictors of resistance have been considered separately; there is therefore a need to revisit these hypotheses in a unified analysis. Here, we do so in the context of a major bacterial pathogen, *Streptococcus pneumoniae*.

In *S. pneumoniae*, both views are supported by large-scale data analyses (*5*, *7*, *8*). A previously proposed deterministic evolutionary ecology perspective of resistance evolution in *S. pneumoniae* explains variation in antibiotic resistance among pneumococcal lineages through variation in the duration of carriage (*5*), which itself is a heritable bacterial trait (*9*, *10*). Theory suggests that the effect resistance has on the fitness of a lineage in species that are mainly carried asymptomatically, such as *S. pneumoniae*, depends on the lineage’s duration of carriage (*5*). This is because these bacteria are primarily exposed to antibiotics prescribed for other infections [“bystander selection”; (*11*)]. The probability of antibiotic exposure is therefore constant, and the per-carriage-episode probability of antibiotic exposure is thus greater for longer carriage episodes. As a result, strains with a long duration of carriage gain more, in terms of relative fitness benefit, from resistance. This model therefore predicts a positive association between resistance and duration of carriage in bacterial species like *S. pneumoniae*. The carriage duration of pneumococcal serotypes is positively correlated with their resistance frequencies in multiple datasets (*5*). The “genetics as limiting factor” hypothesis, on the other hand, predicts a positive association between levels of antibiotic resistance and recombination, an association that is also supported by published data on *S. pneumoniae* (*7*, *12*–*14*). Given these two conflicting theories, each supported by data, we aimed to test both, consistently and within a single dataset.

**Fig. 1:**
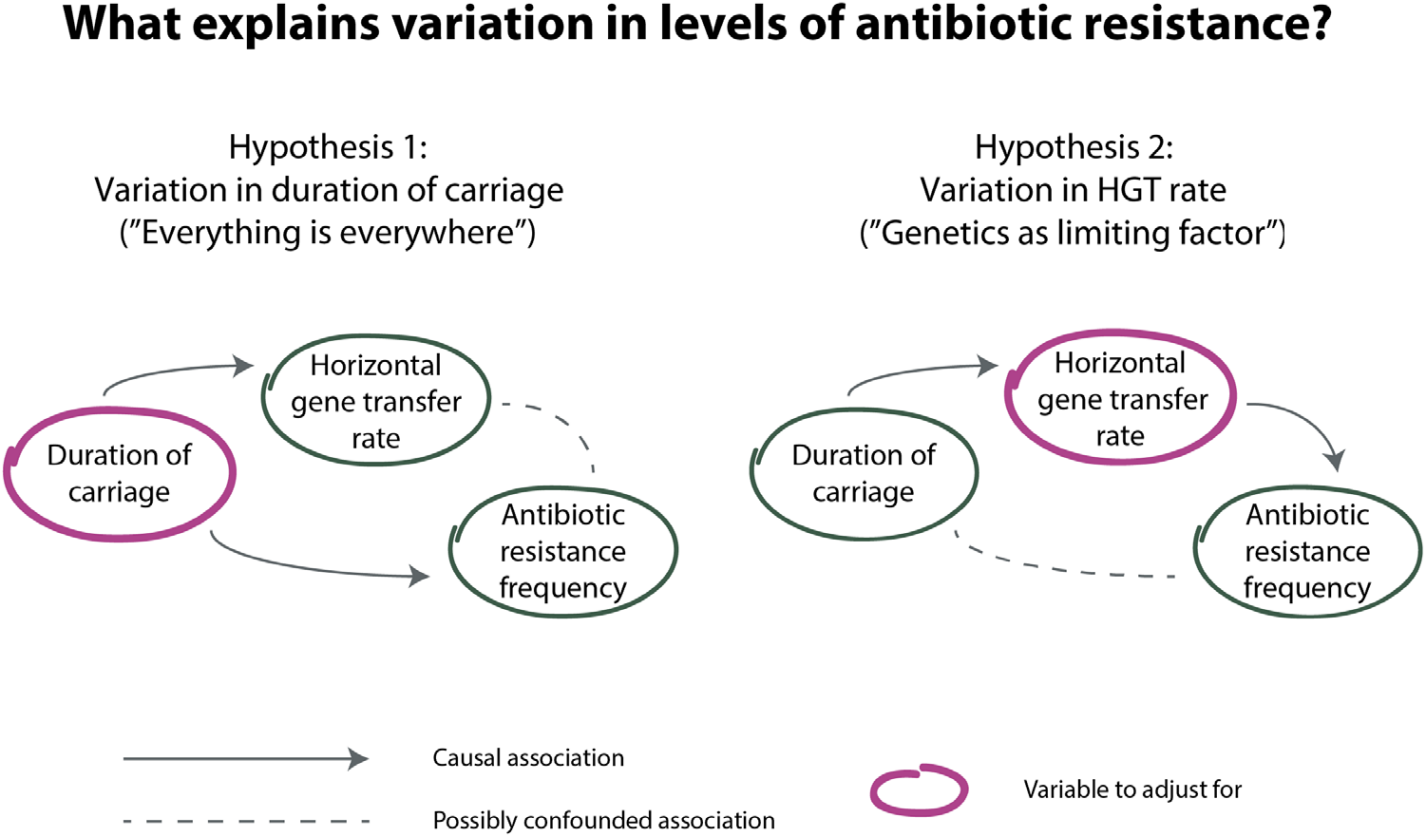
Schematic of the two possible explanations for variation in levels of antibiotic resistance between bacterial lineages. The first hypothesis (“everything is everywhere”; left) is that lineages with a long duration of carriage have high resistance frequencies because resistance is more beneficial to these lineages (because longer duration of carriage translates into a greater probability of antibiotic exposure per carriage episode). The second hypothesis (“genetics as limiting factor”; right) is that lineages with high HGT rate have high resistance frequencies because they acquire resistance determinants at a higher rate. Positive associations have been observed between all three variables (resistance, HGT rate, and duration of carriage). If the first hypothesis is correct, the association between resistance and HGT rate could be confounded by the causal path through the duration of carriage; if the second hypothesis is correct, the association between resistance and duration of carriage could be confounded by the causal path through HGT rate. In the case of a confounding effect, these associations will not be robust to adjusting for the confounded variable.

The interpretation of these published results is complicated by the observation that there is also an association between the duration of carriage and HGT rate. Chaguza *et al.* (*15*) find a positive correlation between the duration of carriage and HGT rate of pneumococcal serotypes. The authors suggest that this is because longer carriage episodes lead to sustained exposure to co-colonizers. HGT requires the presence of the donor and recipient lineages within the same host. Greater exposure to co-colonizers therefore means more opportunities for HGT. The association between the duration of carriage and HGT rate raises the possibility that one of the other two associations could be confounded (Fig. 1). That is, it is possible that the association between resistance and HGT rate is not causal and arises because long duration of carriage causes both high resistance and high HGT rate (supporting “everything is everywhere,” weakening support for “genetics as limiting factor”). Similarly, it is also possible that the association between resistance and duration of carriage is itself not causal and instead arises because long duration of carriage causes high HGT rate, which, in turn, causes high resistance frequency (supporting “genetics as limiting factor,” weakening support for “everything is everywhere”).

Here, using a large pneumococcal isolate collection, which combines microbiological, epidemiological, and genetic data, we investigated the relationship between antibiotic resistance, HGT rate, and duration of carriage in a unified analytic framework. By testing which of the two properties of interest—HGT rate or duration of carriage—is the better predictor of per-lineage antibiotic resistance frequency, we assessed the relative importance of the “everything is everywhere” and “genetics as limiting factor” perspectives in pneumococcal resistance dynamics.

## RESULTS

### Data and definitions

We analyzed a dataset consisting of pneumococcal carriage episodes (*n* = 1179) collected as part of a longitudinal study of pneumococcal carriage in a refugee camp on the Thailand-Myanmar border between 2007 and 2010 (*16*). Carriage episodes were inferred from the longitudinal samples by Lees *et al.* (*10*) using a hidden Markov model. The duration of an episode was defined as the time from colonization with a lineage to clearance of the lineage. Each carriage episode was associated with phenotypic resistance status for seven different antibiotics [obtained from both Chewapreecha *et al.* and Lees *et al.* (*3*, *10*)], and we computed resistance multiplicity for each episode, defined as the number of antibiotics the episode was resistant to (i.e., between zero and seven in this dataset).

HGT rate was computed on the basis of a phylogenetic approach using whole-genome sequences of 2663 isolates from the same pneumococcal population [obtained from Corander *et al.* (*8*)]. Because of the different processes involved in HGT in *S. pneumoniae*, we used two measures aiming to capture two different mechanisms of HGT. The first measure, homologous recombination (HR), aimed to quantify the horizontal transfer of single-nucleotide polymorphisms (SNPs). HR events, typically occurring via transformation, were detected as genomic regions of highly clustered polymorphisms in the context of the phylogeny (*17*). Such clustered SNPs were classified as horizontally acquired, and the HR rate was calculated as the ratio of horizontally to vertically acquired SNPs (commonly known as *r*/*m*). Note that this method detects recombination between lineages: HR between very similar genomes will therefore not be included in our estimates of HR rate. The second measure, gene movement (GM), aimed to quantify the horizontal flow of entire genes, driven by the movement of mobile genetic elements (such as integrative conjugative elements) or illegitimate recombination. This HGT measure was defined as the sum of gene gains or losses per vertical substitution based on ancestral reconstruction of the patterns of gene presence/absence in the phylogeny (*18*). (See Methods for details on both measures.) These two metrics were not correlated [Pearson’s correlation coefficient: −0.09; 95% confidence interval (CI): −0.37 to 0.20], suggesting that they captured different facets of HGT (see the Supplementary Materials).

### Data aggregation

Testing for association at the level of individual carriage episodes is problematic because these carriage episodes may be closely related via recent transmission and may therefore be nonindependent. To circumvent this problem, episodes of carriage need to be aggregated before testing for association. Episodes of carriage could be aggregated by serotype or sequence cluster (SC), i.e., group of closely related sequences. The choice of aggregator is not trivial: Both serotype and SC are potential predictors of our three traits of interest. We therefore used regression models to test which aggregator is the best predictor of the three traits of interest and found that models with SC as the predictor fit the data best (see Table 1 and the Supplementary Materials).

**Table 1:** Akaike information criterion for regression models using serotype, SC, and serotype-SC combination as a predictor of resistance, HGT rate, and duration of carriage. The lowest AIC is indicated in bold.

**Trait**	**Serotype**	**SC**	**Serotype-SC**
Resistance multiplicity	3224	**3163**	3191
Carriage duration	13,243	**13,220**	13,269
HR	4370	4271	**4251**
GM	**88,928**	88,960	88,965

### Correlation with resistance

**Fig. 2:**
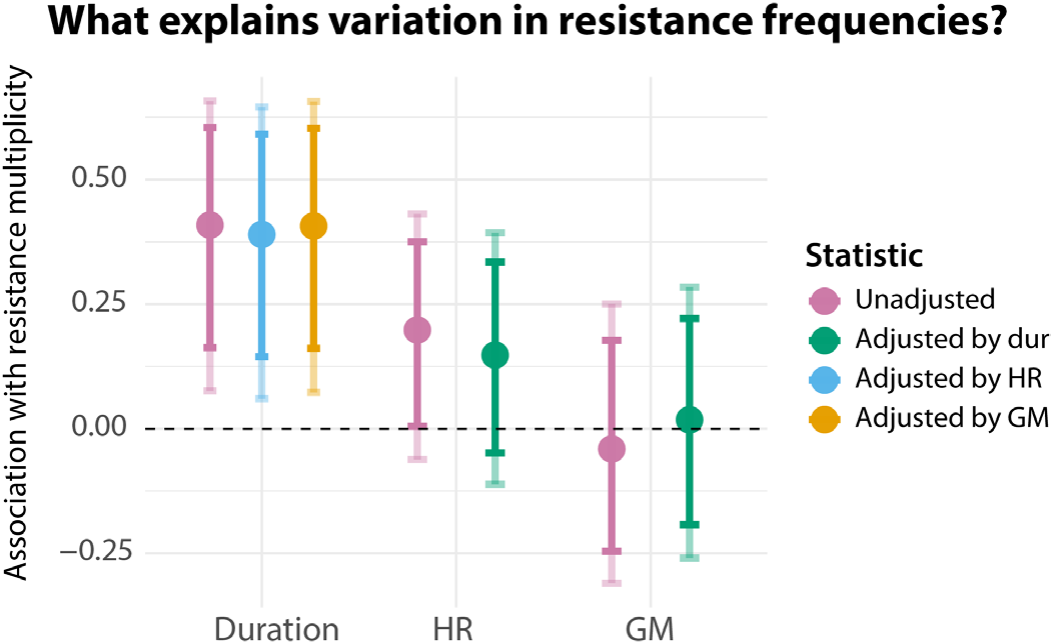
Association with resistance multiplicity. Kendall rank correlation (τ) between SC resistance multiplicity and duration of carriage (unadjusted and adjusted for HR or GM); SC resistance multiplicity and HR (unadjusted and adjusted for duration of carriage); and SC resistance multiplicity and GM (unadjusted and adjusted for duration of carriage).Error bars represent 95% CIs and were computed by bootstrapping (see the Supplementary Materials). SCs with fewer than five episodes of carriage were excluded, giving a sample size of 43 SCs.

We averaged the data by SC (see Methods for details of aggregation and fig. S10 for a visualization of these averages) and computed associations between resistance (both individually against each antibiotic and in terms of resistance multiplicity, i.e., the number of antibiotics an isolate is resistant to) and HGT rate, and resistance and duration of carriage using Kendall’s rank correlation coefficient τ. CIs were generated using bootstrapping (see Methods). We found strong evidence for an association between resistance multiplicity and duration of carriage (τ: 0.41; 95% CI: 0.16 to 0.60), which was robust to adjusting for either measure of HGT rate (Fig. 2). We found no evidence for an association between GM and resistance multiplicity, whether or not adjusting for duration of carriage. We found weak evidence for an association between resistance multiplicity and HR (τ: 0.20; 95% CI: 0.00 to 0.38), which became nonsignificant when adjusting for duration of carriage. These results were qualitatively robust to changes in the parameters underlying estimation of HGT rate and classification of intermediate resistance (see Methods and fig. S9). The strength of the association between HR and resistance was sensitive to the extent to which long phylogenic branches were included in the estimation of HGT rates (normally excluded because of the large uncertainty associated with the inference of HGT on long branches). Inclusion of very long branches led to the strengthening of the unadjusted association between HR and resistance, but the adjusted association remained nonsignificant (see Methods for further discussion).

**Fig. 3:**
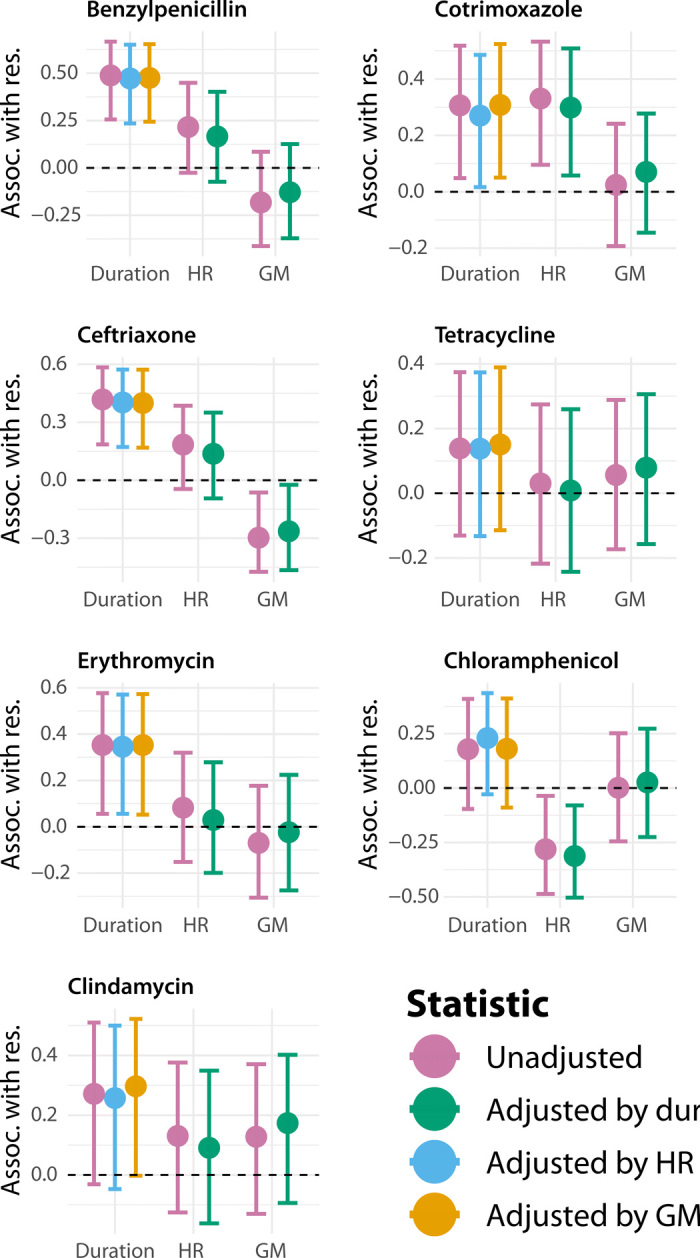
Association with individual resistances. Kendall rank correlation (τ) between SC resistance to each antibiotic and duration of carriage (unadjusted and adjusted for HR or GM); SC resistance to each antibiotic and HR (unadjusted and adjusted for duration of carriage); and SC resistance to each antibiotic and GM (unadjusted and adjusted for duration of carriage). Opaque error bars represent 95% CIs, while transparent error bars represent 99% CIs; both were computed by bootstrapping (see the Supplementary Materials). SCs with fewer than five episodes of carriage were excluded, giving a sample size of 42 SCs.

Similar trends held when analyzing antibiotics individually (Fig. 3). In general, resistance was more strongly associated with the duration of carriage than either measure of HGT rate, and this association was undiminished when adjusting for HGT. Cotrimoxazole was an exception to this pattern: Cotrimoxazole resistance was more strongly associated with HR than duration of carriage, and this relationship remained statistically significant when adjusting for the duration of carriage. This is consistent with previous observations that cotrimoxazole resistance emerges via HR-mediated acquisition of alleles (*19*), and the corresponding genes had been found to be recombination hotspots (*3*).

Results of the analysis were qualitatively similar when using serotype or serotype-SC as the analysis aggregators (see figs. S3 to S6). First, we found strong evidence for an association between resistance multiplicity and duration of carriage, even when adjusting for either measure of HGT. Second, we found no evidence for an association between resistance multiplicity and GM, whether adjusting for the duration of carriage or not. The evidence for an association between resistance multiplicity and HR was mixed: For both serotype and serotype-SC, HR was associated with resistance multiplicity in the unadjusted analysis. When adjusting for the duration of carriage, the association weakened in both cases but remained statistically significant when using serotype-SC as the aggregator (τ: 0.18; 95% CI: 0.01 to 0.33).

Last, we did not replicate a significant correlation between HGT rate and duration of carriage (*15*) for either of our measures of HGT rate (τ: 0.15; 95% CI: −0.11 to 0.40 for HR; τ: −0.14; 95% CI: −0.37 to 0.11 for GM). These associations were also nonsignificant when aggregating by serotype and serotype-SC.

## DISCUSSION

Our study aimed to discriminate between the “everything is everywhere” and “genetics as limiting factor” views of antibiotic resistance evolution by testing the relationship between resistance, rates of HGT, and duration of carriage. Together, these results provide strong support for an association between the duration of carriage and resistance, which is robust to adjusting for the rate of HGT. Conversely, we found only weak evidence for an association of HR and resistance, which weakened further when adjusting for the duration of carriage; we found no evidence for an association between GM and resistance. Our data thus do not support a major role for HGT rate in determining the frequency of antibiotic resistance in pneumococcal lineages, hence lending support to the deterministic eco-evolutionary perspective (“everything is everywhere”).

This work was motivated by the reported correlations between resistance, HGT rate (specifically HR), and duration of carriage. We only replicate a significant correlation for resistance and duration of carriage—our point estimates for the other two correlations are positive but generally nonsignificant. One possible explanation for this discrepancy could be differences in the way HR is estimated. This is unlikely to be the case, however, as our estimates are strongly correlated with previously used measures (fig. S1). Another potential explanation is that our treatment of uncertainty is more conservative than in previous work. Our CIs take into account the uncertainty in estimating the per-cluster averages. This is particularly pertinent for HR, because recombination rates are often based on a small number of recombination events, leading to large CIs and thus potentially explaining why we observe positive, but nonsignificant, associations between HR and resistance and HR and duration of carriage (fig. S2). Computing the correlation between HR and duration of carriage without accounting for uncertainty in the per-cluster estimates gives a higher, although still negative, lower bound for the 95% CI (table S2).

Our results provide strong evidence against a role of GM in determining resistance frequencies. The evidence regarding the role of HR is less clear. Point estimates for the association between HR and resistance were consistently positive (although generally nonsignificant), even when adjusting for the duration of carriage. Given our conservative treatment of uncertainty in the estimates of HR, these results do not rule out a potential independent role for HR, or some other correlate of HR, in determining resistance frequencies. A potential causal role for HR is supported by the observation that the magnitude of the correlation between HR and resistance was greatest for the resistance determinants that are not carried on transposons and would therefore be expected to be acquired through HR rather than GM (benzylpenicillin, ceftriaxone, and cotrimoxazole resistance). On the other hand, the acquisition of benzylpenicillin resistance requires changes in multiple genes and therefore likely multiple HR events (*3*), while cotrimoxazole requires only one (*19*). If HR is a rate-limiting step in the evolutionary dynamics of resistance, we might expect the HR rate to have a greater impact on resistance frequencies when multiple steps are required. This is not what is observed: The association between HR and resistance is lower for benzylpenicillin than for cotrimoxazole. Overall, although HR rate cannot be ruled out as a driver of resistance frequencies, the small magnitude of the association between HR and resistance suggests that this role is relatively minor compared to the role of duration of carriage.

An interesting aside to our analysis is the choice of aggregation, by SC, serotype, or both (Table 1). While this was performed for methodological reasons, it provides some additional insights into the genetic correlates of our phenotypes of interest, suggesting, in particular, that both duration of carriage and resistance are more closely associated with SC than with capsular serotype.

There are some caveats to our conclusions that are worth highlighting. First, we have interpreted the robustness of the association between duration of carriage and resistance as supporting the role of bacterial duration of carriage as a major driver of antibiotic resistance. An important caveat here is that there is uncertainty with respect to the direction of causality between resistance and duration of carriage; such association could also arise because resistance prevents clearance through antibiotic exposure, thus prolonging the duration of carriage. We attempted to establish whether the association between resistance and carriage duration was still present when eliminating the effect of resistance on the duration of carriage, but our results were inconclusive (presumably due to insufficient statistical power; see Supplementary text). However, as some of us have previously suggested (*20*), the association being observed at the serotype level lends support to the role of duration of carriage in determining resistance frequencies. Second, resistance in this dataset was measured in laboratory assays. From an evolutionary perspective, the most relevant definition of resistance is increased transmission potential in the presence of antibiotics; resistance, as defined in the laboratory, may not fully correspond to this evolutionary definition. Furthermore, neither the laboratory nor the evolutionary definition fully corresponds to the definition of clinically relevant resistance, so care should be taken when interpreting these results in a public health context.

Overall, our analysis supports an “everything is everywhere” view of pneumococcal resistance evolution, in which observed allele frequencies and combinations depend on the fitness of these genotypes and are not constrained by a history of genetic events. Yet, the role of HGT in acquisition of resistance determinants is well documented (*2*, *3*, *19*, *21*, *22*). This apparent contradiction can be resolved by placing our results in the context of the time frame of this study. Antibiotic use has been present over a time scale of several decades, and during the course of this study, resistance frequencies were not observed to change (see fig. S7), suggesting that they were probably at quasi-equilibrium. The picture that emerges, therefore, is that while HGT rate plays a role in how long it takes for a pneumococcal population to acquire novel resistance determinants, the eventual frequency of these determinants can be understood purely in terms of evolutionary optimization. Our study leaves open the question of the role of genetic processes in nonequilibrium populations. The studies that have reported a correlation between HGT and resistance (*7*, *12*–*14*) spanned different spatial and temporal scales and may therefore have sampled nonequilibrium pneumococcal populations. Public health measures such as vaccination programs or changes in treatment regimes can be viewed as perturbations that change the fitness landscape of the pathogen population. Addressing the relative importance of genetic and selection processes in the short-term dynamics following such a perturbation is therefore necessary to predict the effect of medical interventions on bacterial populations.

Last, whether this picture of resistance evolution also applies to species other than *S. pneumoniae* remains to be established. In other species, differences in ecology and HGT mechanisms may lead to differences in the relative importance of HGT and selective pressure in determining resistance frequencies. In particular, when antibiotic resistance is associated with plasmids, plasmid dynamics and plasmid competition may affect the relationship between HGT rate and resistance frequencies. However, while the specific results we report may not generalize beyond *S. pneumoniae*, the distinction between the “everything is everywhere” and “genetics as limiting factor” views does. Recognizing this distinction between these perspectives in other species as well is a key step in developing a cohesive theory of the evolutionary dynamics of antibiotic resistance.

## METHODS

### Episodes of carriage: Duration, resistance, and cluster assignment

#### The pneumococcal carriage study

Data used in this analysis were collected as part of a longitudinal study of pneumococcal carriage in infants and their mothers in a 4-km^2^-area refugee camp on the Thailand-Myanmar border area between 2007 and 2010, where the pneumococcal vaccine was not available (*16*, *23*). Briefly, the study recruited 999 pregnant women and followed all 955 live births until their second birthday. Nasopharyngeal swabs were taken monthly from all infants and from a subset of 234 mothers. All swabs from the 234 mother-infant pairs, as well as selected swabs from the remaining infants (those taken during or immediately before a clinical pneumonia episode), were cultured and serotyped according to the World Health Organization protocol as described in (*16*) and phenotypically tested for susceptibility to seven classes of antibiotics (benzylpenicillin, ceftriaxone, cotrimoxazole, tetracycline, erythromycin, chloramphenicol, and clindamycin) and classified as susceptible, intermediate, or resistant according to the Clinical and Laboratory Standards Institute criteria (2007). Intermediate isolates were considered resistant. A random subset of these samples were subsequently whole genome–sequenced, as described in (*3*). These data (*n* = 2663 genome assemblies) were obtained from Corander *et al.* (*8*). In addition, all swabs from another 364 infants (i.e., infants not part of the 234 mother-infant pairs) were serotyped using the latex sweep method as described in (*24*).

#### Duration of carriage estimates

The two longitudinal datasets (i.e., swabs from the 234 infants and 364 infants) had previously been used to estimate the duration of different episodes of carriage by fitting a hidden Markov model to the data of which serotypes were detected at each time point (*10*). Note, therefore, that the inference of these carriage episodes was based on continuous carriage of the same serotype rather than the same genetic sequence. These inferred carriage episodes (*n* = 1331 episodes) and their estimated duration were obtained from Lees *et al.* (*10*).

#### Serotype and cluster assignment

For the sequence assemblies obtained from Corander *et al.* (*8*), we used the pipeline previously described in Mostowy *et al.* (*25*) to infer serotype based on genetic sequence. Sequence quality was too low to infer serotype for four of the isolates; these isolates were assigned the experimentally characterized serotype. For a small number of swabs (77), the experimentally determined serotype was different from the sequence-derived serotype; for these, we used the sequence-derived serotype for further analysis. SC assignments were obtained from Corander *et al.* (*8*). These had been determined using a species-wide clustering analysis based on genetic similarity combining four major datasets. To obtain reliable estimates of HGT rate (see below), SCs with below 10 isolates were excluded from further analysis, leaving 48 of 55 SCs.

#### Combining datasets

Our data were therefore made up of two datasets: an epidemiological dataset consisting of a set of carriage episodes, each associated with a duration, a serotype, and resistance status for seven classes of antibiotics; and a genetic dataset, consisting of sequences originating from a random subset of these carriage episodes (with some episodes having been multiply sequenced), each associated with a serotype and SC assignment. Combining the genetic and epidemiological datasets gave a set of carriage episodes (*n* = 1179) with associated duration, serotype, resistance to seven classes of antibiotics and SC assignment. When multiple sequences originated from the same carriage episode, these were considered as a single data point (i.e., the carriage episode was not counted twice in the analysis). For the majority of carriage episodes with multiple sequences, the sequences had identical properties (i.e., same serotype, SC assignment, and resistance status). On occasion, sequences from the same carriage episode had inconsistent serotypes (7 carriage episodes) or SC assignments (14 carriage episodes). Inconsistency in serotype was due to inconsistency between genetically and experimentally inferred serotype. Inconsistency in SC assignment was due to episodes of carriage having been inferred on the basis of serotype only: Two consecutive episodes of carriage of genetically distinct strains with the same serotype would have been inferred to be a single episode of carriage. These inconsistent episodes of carriage were removed, along with carriage episodes from clusters with fewer than 10 isolates (see above), giving a dataset of 1091 carriage episodes. For 145 of these, sequences from the same episode of carriage had consistent SC assignments but different resistance profiles. We assumed that this represented within-host evolution rather than infection with distinct strains. For these episodes of carriage, we used the mean value of the resistance statuses (e.g., if the episode consisted of one resistant and one susceptible isolate, we used 0.5 as the resistance status).

#### HGT rate

HGT, broadly defined as horizontal transfer of genetic information, was quantified, independently for each SC, using two metrics. The first metric, called HR, was the proportion of genetic variation (SNPs) acquired horizontally to genetic variation acquired vertically, commonly known as *r*/*m*. The second metric, called GM, captures the rate of gene gain or loss in the SC. In broad terms, we obtained these measures by first defining a core genome for each SC, then using a phylogenetic approach to recognize clusters of SNPs as HR events in this core genome tree, and then combining the core genome tree with the absence/presence of accessory genes in the sampled genomes to identify gene gain and loss events.

In more detail, we first defined a core genome for each SC. To achieve this, we first predicted protein coding sequences in 2663 genome assemblies [using Prodigal (*26*)] and assigned these sequences into clusters of orthologous genes (COGs) [using mmseqs with default parameters and --min-seq-id 0.5 option (*27*)]. We defined the core genome for each SC as a set of COGs in a given SC that are uniquely present in at least 70% of all isolates in that SC. These COGs were then concatenated into a core genome alignment in a random order (see below). Next, we detected horizontally acquired SNPs on these core genomes using Gubbins (*17*) (with default parameters), a software that uses a phylogenetic approach to assign SNPs into those acquired horizontally and vertically and to return a clonal phylogeny. Next, we inferred the number of gains and losses of the remaining COGs on each branch of this clonal phylogeny using GLOOME (*18*), which uses a stochastic mapping approach to estimate the horizontal gene flow given a gene presence/absence profile and a phylogenetic tree. The end result of this analysis was a table where each row showed an independent branch of a clonal tree in a given SC, the number of SNPs acquired via mutation and recombination, the number of gene gains and losses, and the branch length following a removal of horizontally acquired variation. The longest branches of the tree (5000 SNPs or longer) were excluded because of large uncertainty in recombination estimates at those branches (*17*). The HR rate was defined as the total sum of horizontally acquired SNPs to the sum of vertically acquired SNPs. The GM rate was defined as the total sum of gene gains and losses divided by the total branch length of the clonal tree. The CIs were obtained by resampling branches of the tree with replacement *n* = 1000 times for each SC.

To validate our pangenome-based method of inferring HR rates, we applied this method to *n* = 616 isolates from a Massachusetts pneumococcal collection for which *r*/*m* rates were obtained by mapping each SC to a draft pneumococcal genome (see fig. S1). Results show that, while there was a strong concordance between the two measures (*R*^2^ = 0.89; 95% CI: 0.44 to 0.98), there was a small discrepancy between the two measures for some SCs. To determine whether this discrepancy was due to a false detection rate of HGT stemming from suboptimal concatenation of COGs in the core alignment, we investigated the effect of random COG concatenation on the estimation of HR and GM rates (see fig. S2). We found that the uncertainty in estimates of both HR and GM rate was comparable or smaller than the rate uncertainty obtained by branch resampling, suggesting that the COG concatenation had a limited influence on the estimate of HGT rates. We thus concluded that the observed discrepancies between the estimates of *r*/*m* using our pangenome approach and those approaches using a full reference sequence are mostly driven by the GM of low-to-intermediate frequency genes in the reference sequence.

Last, to confirm that our estimates of HGT rate were not driven by transfer events involving resistance genes (in which case, high HGT rates in a particular lineage might simply have reflected higher selection pressure for resistance), we reestimated HR and GM rates having removed antibiotic resistance determinants (based on sequence similarity to resistance determinants in the Antibiotic Resistance Genes Database) (*28*)). These estimates were strongly correlated with the estimates in the main analysis (fig. S8), suggesting that observed HGT rates are not driven by the acquisition of resistance.

#### Choice of aggregator

The choice of aggregator was based on fitting regression models to determine whether serotype, SC, or serotype-SC best predicted the traits of interest (HGT rate, duration of carriage, and resistance status). For each combination of potential aggregator and trait of interest, we fitted a regression model with the aggregator (i.e., serotype, SC, or serotype-SC) as a categorical predictor variable and the trait (HGT rate, duration of carriage, or resistance multiplicity) as an outcome variable. For resistance multiplicity and duration of carriage, which are properties of a carriage episode, the models (Poisson for resistance multiplicity and linear for duration of carriage) were fitted to the carriage episode dataset. For the HGT rate metrics, the models (logistic for HR and linear for GM) were fitted to the phylogeny branches. The quality of the fits was assessed using Akaike information criterion (AIC) [$-2\text{ln}(\hat{L})+2k$], where $\hat{L}$ is the maximum value of the model’s likelihood function and *k* is the number of parameters to fit (48, 48, and 86 for serotype, SC, and serotype-SC, respectively in the carriage episode dataset).

#### Correlation with resistance

Associations between SC mean resistance and SC mean recombination rate, and SC mean resistance and SC mean duration of carriage, were tested using Kendall’s rank correlation tau (τ). Kendall’s tau was chosen as a metric (rather than Spearman’s rho) because of the nature of uncertainty in our data: Clusters with fewer samples are likelier to have extreme resistance values (i.e., 0 or 100% resistance), and Kendall’s tau is less sensitive to error at the extremes of the distribution than Spearman’s rho. The adjusted associations between resistance and recombination rate controlling for carriage duration and resistance and carriage duration controlling for recombination rate were calculated using partial Kendall’s rank correlation as implemented in ppcor R package (*29*).

Calculating the correlation using SC means would not account for the uncertainty in the estimation of these means. To address this, we used a dual-bootstrap approach to compute CIs for tau: If *P*(τ∣θ) is the sampling distribution of tau, given the SC properties to correlate (θ, e.g., resistance and duration of carriage), and *P*(θ) is the sampling distribution of the SC properties, then the overall sampling distribution for *P*(τ) is *P*(τ) = ∫_θ_*P*(τ∣θ)*P*(θ)*d*θ. Both *P*(τ∣θ) and *P*(θ) were estimated through bootstrapping using the following procedures:

1) For each SC, draw, with replacement, from carriage episodes in the SC, a set of carriage episodes of the same size as the SC. Calculate the mean carriage duration.
2) Similarly, for each SC, draw a set of resistance multiplicities and calculate mean resistance multiplicity.
3) For each SC, draw, with replacement, from the SC phylogeny, a set of random branches the same size as the number of branches in the SC phylogeny. Calculate mean HR and HGT.
4) Sample with replacement among these SC estimates, calculate τ, repeat *m*_b_ times to give estimate of *P*(τ∣θ) for a particular θ.
5) Repeat steps 1 to 4, *n*_b_ times.
6) Sum the *n* distributions to give *P*(τ).

Ninety-five percent CIs were calculated from this distribution. SCs with fewer than five observations were excluded from the analysis. The bootstrap samples used are *n*_b_ = *m*_b_ = 1000 unless stated otherwise.

#### Sensitivity analysis

In addition to the sensitivity analysis discussed in Results (sensitivity to the choice of aggregator; figs. S3 to S6), we also performed additional sensitivity analyses for key steps in our data processing. First, classification of intermediate resistance (as resistant in the main analysis and as sensitive in the sensitivity analysis); second, the cutoff for exclusion of long branches from the analysis in computing HGT rates (5000 SNPs in the main analysis and 1000 and 10,000 in the sensitivity analysis); third, definition of core genome (GOCs present in at least 70% of isolates in the main analysis and 99% of isolates in the sensitivity analysis). Our conclusions (strong evidence for an association between the duration of carriage and resistance, weaker evidence for an association of smaller magnitude between HR and resistance, and no evidence for an association between GM and resistance) were robust to these changes (fig. S9). It is worth noting that the association between HR and resistance was weaker using the 1000 SNP cutoff and stronger when using the 10,000 SNP cutoff). Because of the uncertainty associated with HGT inference for long branches (*17*), the interpretation of this effect is unclear: While the inclusion of these branches allows the inclusion of increased genetic diversity and thus more recombination events, differentiating between horizontal and vertical transmission on long branches is more difficult, thus leading to increased uncertainty in the estimates of HGT rate (which cannot be taken into account in the computation of CIs).

## Supplementary Material

aaz6137_SM.pdf

aaz6137_Tables_S1_and_S2.xlsx

## SUPPLEMENTARY MATERIALS

Supplementary material for this article is available at http://advances.sciencemag.org/cgi/content/full/6/21/eaaz6137/DC1

## Appendix

View/request a protocol for this paper from *Bio-protocol*
