# Supplementary material for: Horizontal gene transfer rate is not the primary determinant of observed antibiotic resistance frequencies in Streptococcus pneumoniae
Source: Sci Adv. 2020 May 20;6(21):eaaz6137. doi: 10.1126/sciadv.aaz6137 (PMC7314567; doi:10.1126/sciadv.aaz6137)
Supplement: aaz6137_SM.pdf [file aaz6137_SM.pdf]

Science Advances
AAAS

advances.sciencemag.org/cgi/content/full/6/21/eaaz6137/DC1

Supplementary Materials for

Horizontal gene transfer rate is not the primary determinant of observed antibiotic resistance frequencies in *Streptococcus pneumoniae*

Sonja Lehtinen, Claire Chewapreecha, John Lees, William P. Hanage, Marc Lipsitch, Nicholas J. Croucher, Stephen D. Bentley, Paul Turner, Christophe Fraser*, Rafał J. Mostowy

*Corresponding author. Email: christophe.fraser@bdi.ox.ac.uk

Published 20 May 2020, *Sci. Adv.* **6**, eaaz6137 (2020)
DOI: 10.1126/sciadv.aaz6137

The PDF file includes:

Supplementary Text
Legends for tables S1 and S2
Figures S1 to S10

Other Supplementary Material for this manuscript includes the following:

(available at advances.sciencemag.org/cgi/content/full/6/21/eaaz6137/DC1)

Tables S1 and S2

Supporting Information

1 Supplementary Text

1.1 Direction of causality for resistance and duration of carriage

As discussed in the main text, we expect a long duration of carriage to be predictive of resistance because resistance is more advantageous in lineages with a long duration of carriage. However, we also expect long duration of carriage to be associated with resistance because resistant strains are less likely to be cleared through antibiotic exposure. To test whether this reverse causality accounts for the association we observe, we compute per-SC carriage duration using only episodes of carriage with the same resistance profile. This eliminates the effect of between-SC variation in resistance frequency on duration of carriage. We use the most common resistance profile (resistance to cotrimoxazole and sensitivity to all other antibiotics, $n = 188$ carriage episodes), which reduces the number of SCs for which we can compute the duration of carriage, to $n = 11$. We find a positive, but no longer significant association between resistance and duration of carriage (**τ: 0.25, 95%CI [-0.42,0.76]**).

The loss of significance might be due to the smaller number of SCs (as opposed to the elimination of the effect of resistance on duration of carriage). To check for this, we look at the correlation between resistance and duration of carriage for the same set of 11 SCs, this time using the duration of carriage estimates from the main analysis (i.e. without restricting to a single resistance profile). Again, we see a positive, but non-significant association **(τ: 0.41, 95%CI [-0.23,0.86])**. This suggests that the loss of the significance is at least partially due to the decrease in sample size. We therefore cannot confidently conclude whether the association between resistance and duration of carriage is driven by the effect of resistance on duration of carriage, the effect of duration of carriage on resistance, or a combination of the two.

2 Supplementary Tables

Table S1. Akaike Information Criterion for logistic regression model using serotype, cluster and serotype-SC combination as a predictor of resistance, HGT rate and duration of carriage (see Methods for details) for resistance against individual antibiotics. The lowest AIC is indicated in bold. The table can be found in a separate CSV file.

Table S2. SC averages for resistance multiplicity, individual resistance, duration of carriage, HR and GM, excluding SCs with fewer than 5 episodes of carriage. The table can be found in a separate CSV file.

3 Supplementary Figures

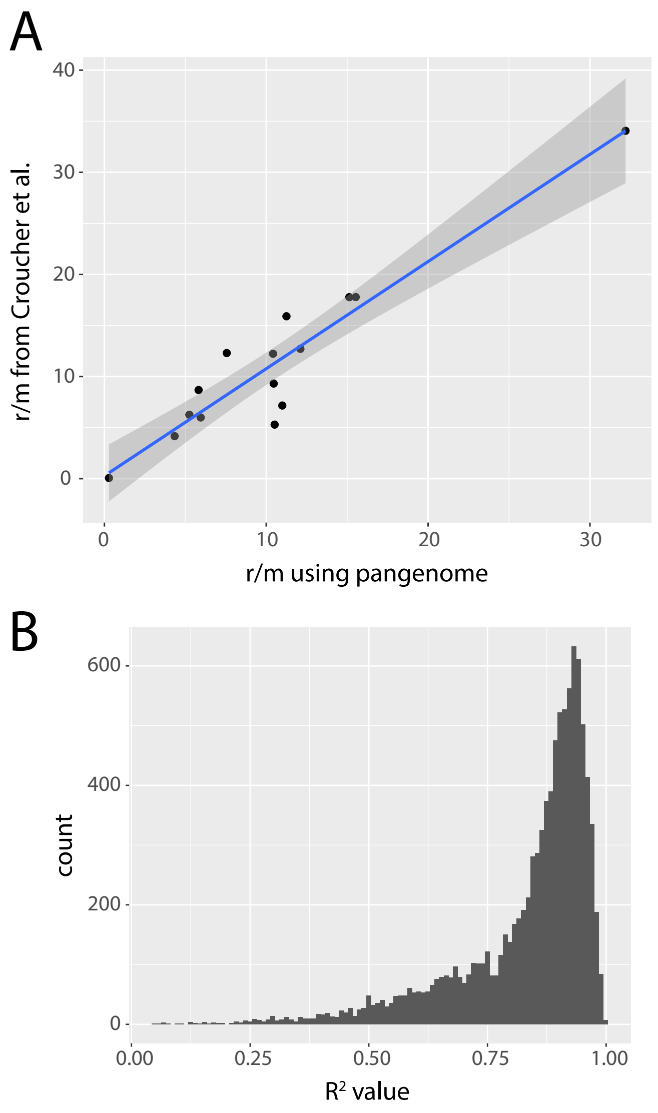

Figure S1. Validity of pangenome-based measure of homologous recombination. (A) X-axis shows the HR (r/m) rate obtained as described in the text for $n = 15$ SCs from Croucher et al. (main text ref 21), while Y-axis shows the original estimates obtained by mapping short-reads to 15 corresponding reference draft genomes. (B) Histogram of R^2 values obtained by resampling 15 points in panel A with replacement $n = 1000$ times and reestimating the correlation coefficient. The resulting correlation between estimates from the two approaches is $R^2 = 0.89$, 95% CI: [0.44,0.98].

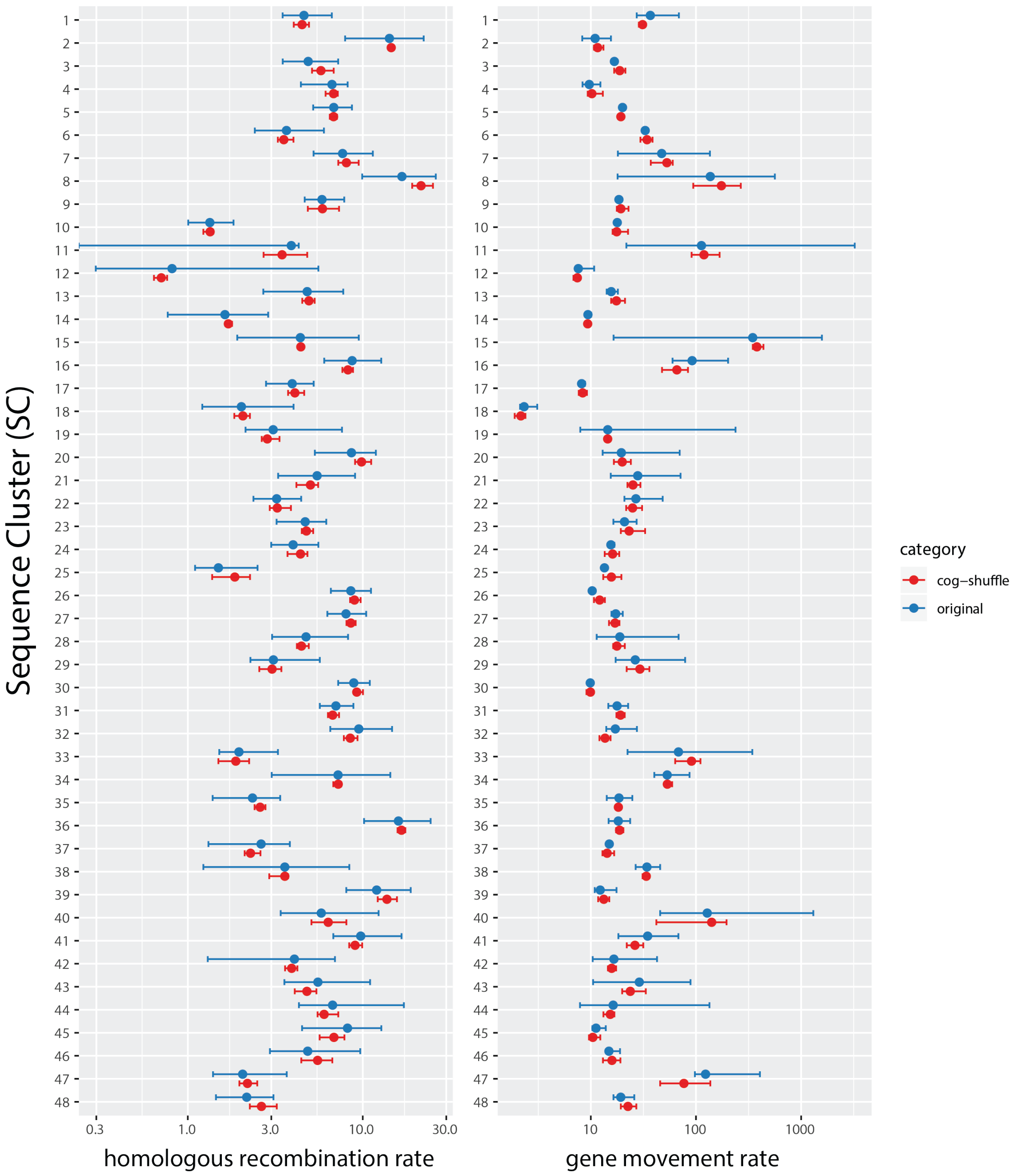

Figure S2. Effect of random order COG concatenation on the estimation of HR rate (left) and GM rate (right). Blue points show the main estimates for all 48 sequence clusters (SC), with error bars showing the 95% confidence intervals obtained by bootstraping branches of the clonal tree, as described in the Supplementary Text. Red error bards and points show the full range of HR/GM values and their median, respectively, obtained by shuffling COGs randomly.

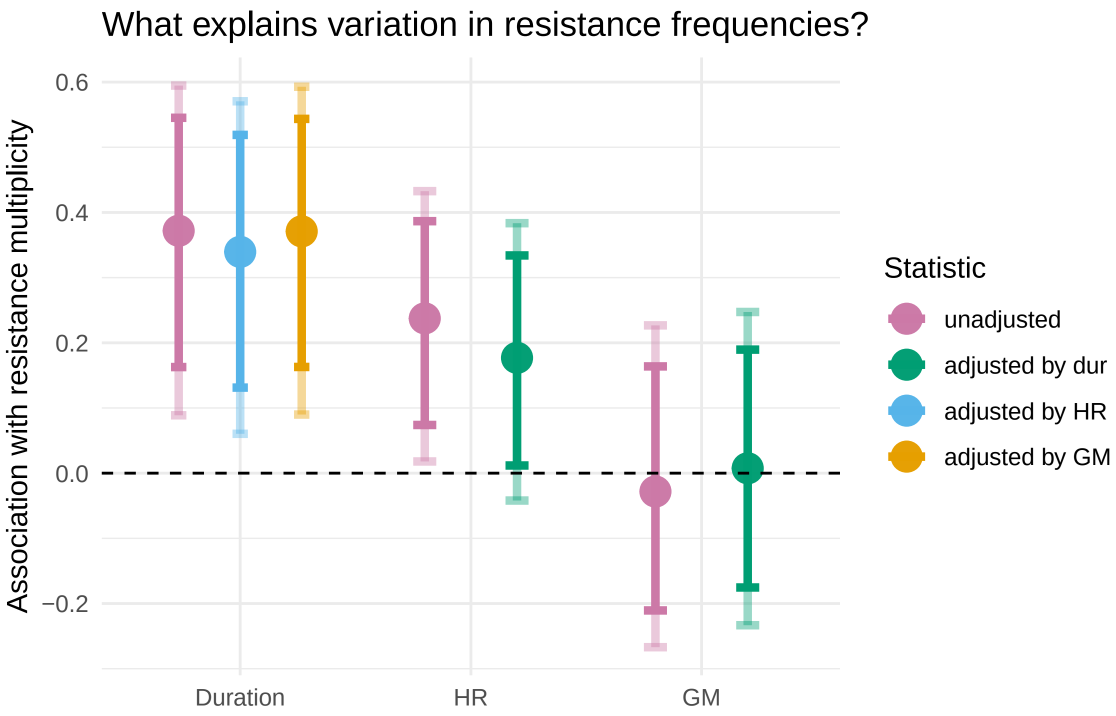

Figure S3. Association between resistance and duration of carriage and two measures of HGT, with serotype-SC as the main unit of analysis. The legend of each panel is the same as described in Figure 2.

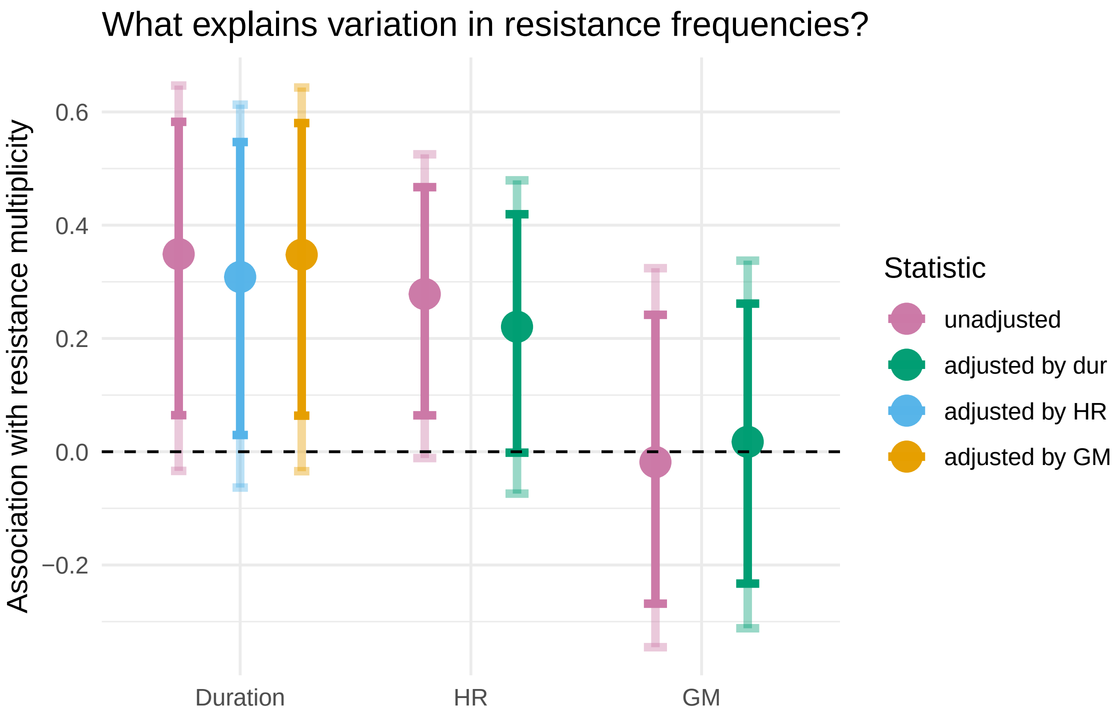

Figure S4. Association between resistance and duration of carriage and two measures of recombination, with serotype as the main unit of analysis. The legend of each panel is the same as described in Figure 2.

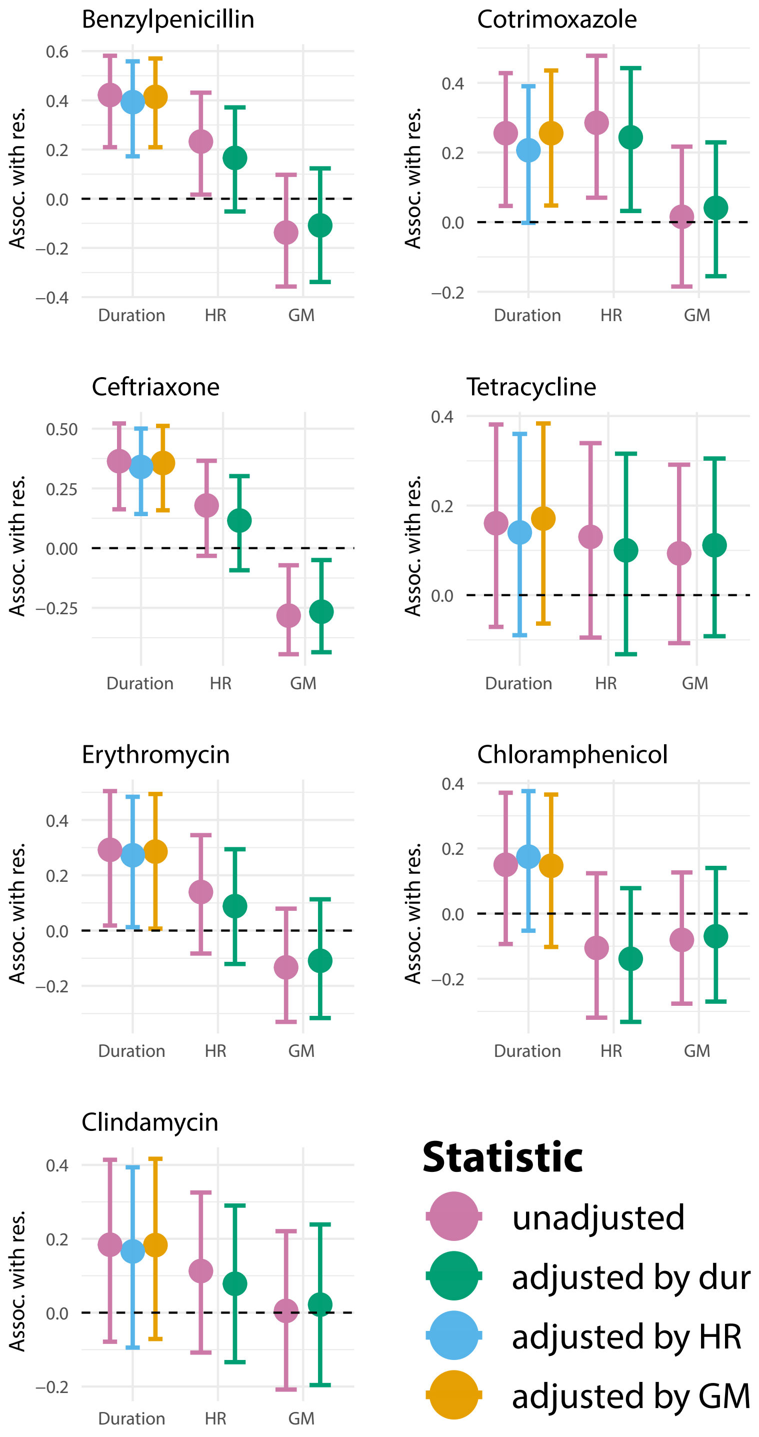

Figure S5. Results for individual classes of antibiotics with serotype-SC as the main unit of analysis. The legend of each panel is the same as described in Figure 2. Number of bootstrap samples is $n_b = m_b = 100$.

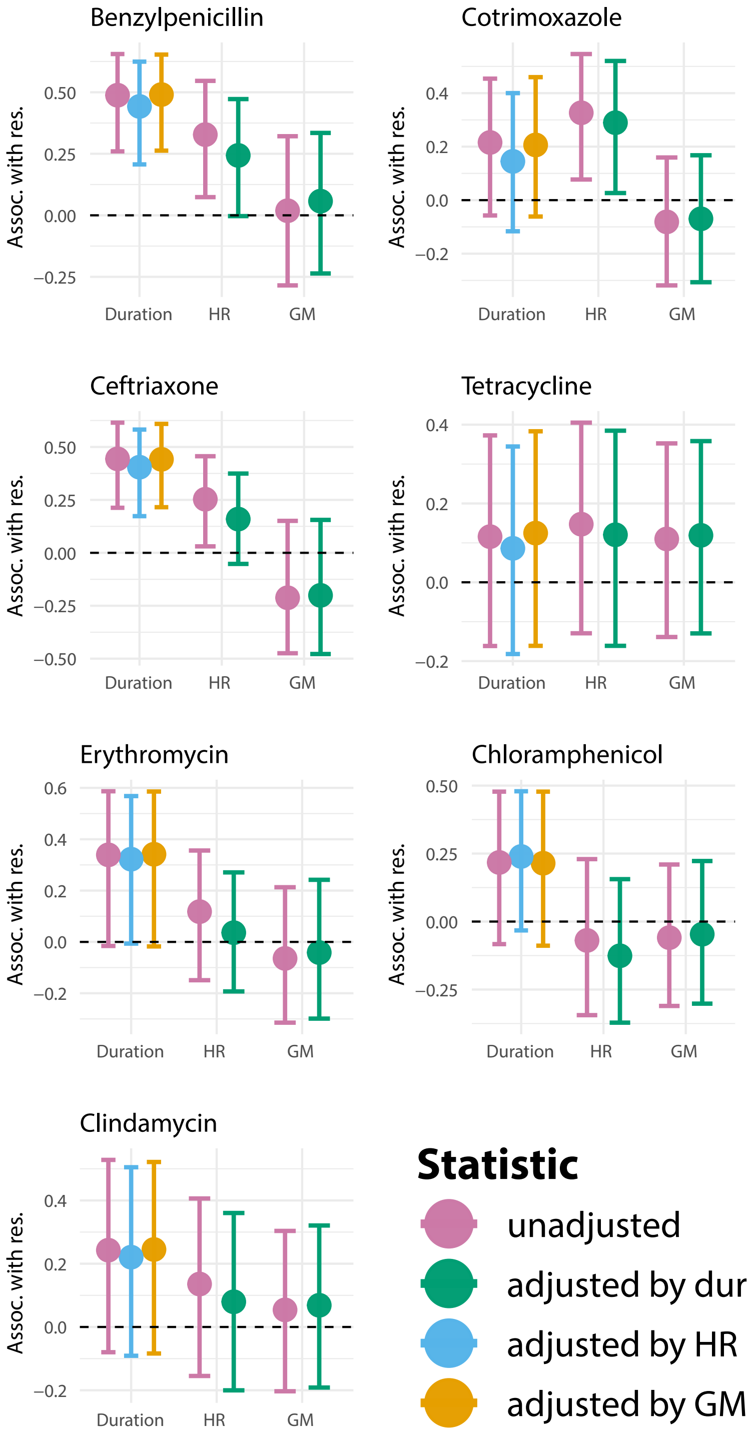

Figure S6. Results for individual classes of antibiotics with serotype as the main unit of analysis. The legend of each panel is the same as described in Figure 2. Number of bootstrap samples is $n_b = m_b = 100$.

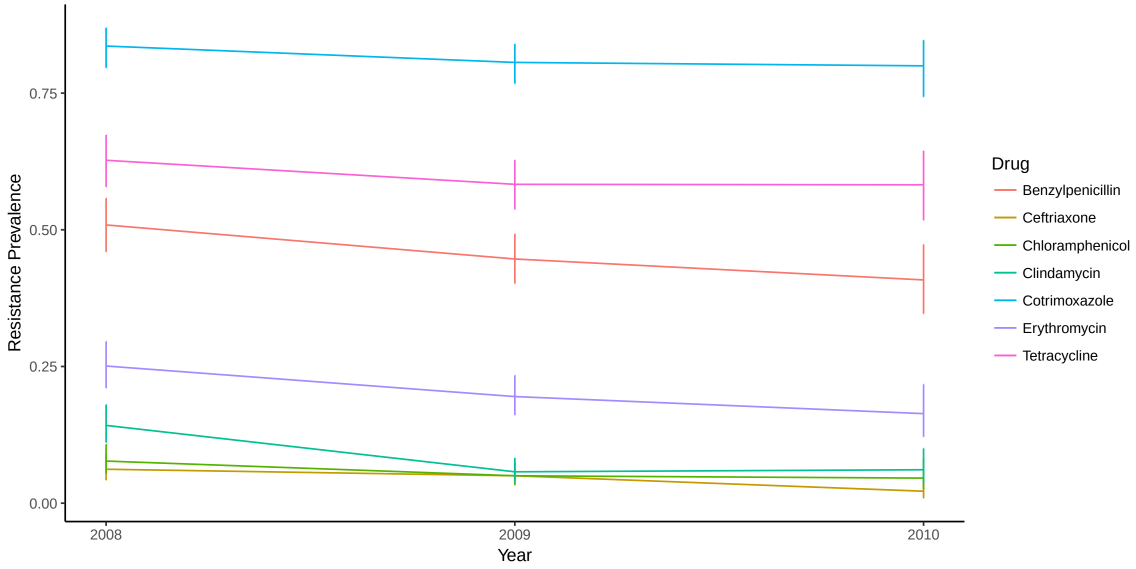

Figure S7. The prevalence of resistance against the seven different antibiotics as a function of time in the Maela population. Error bars represent 95% confidence intervals. For clarity, data from 2007 has not been plotted as only 6 carriage episodes were available.

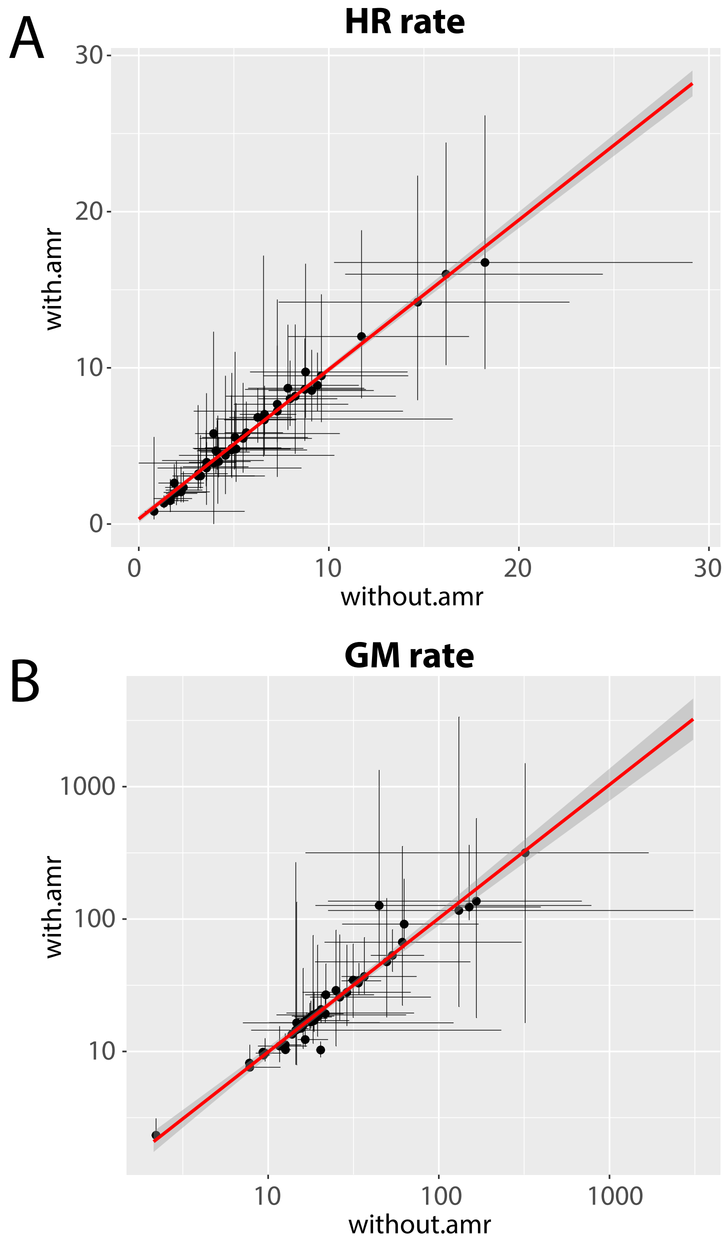

Figure S8. Effect of removing genetic determinants of antibiotic resistance on estimates of the HR rate (A) and GM rate (B). Y-axis shows the values estimated in the main analysis, with the 95% confidence intervals, while X-axis shows the values re-estimated having removed antibiotic resistance determinants. Such determinants were defined as COGs with representatives which gave sequence similarity (`blastp`) hits to the reference proteins defined by the ARDB database (main text ref 28), using e-value of 10^{-10} as a cut-off.

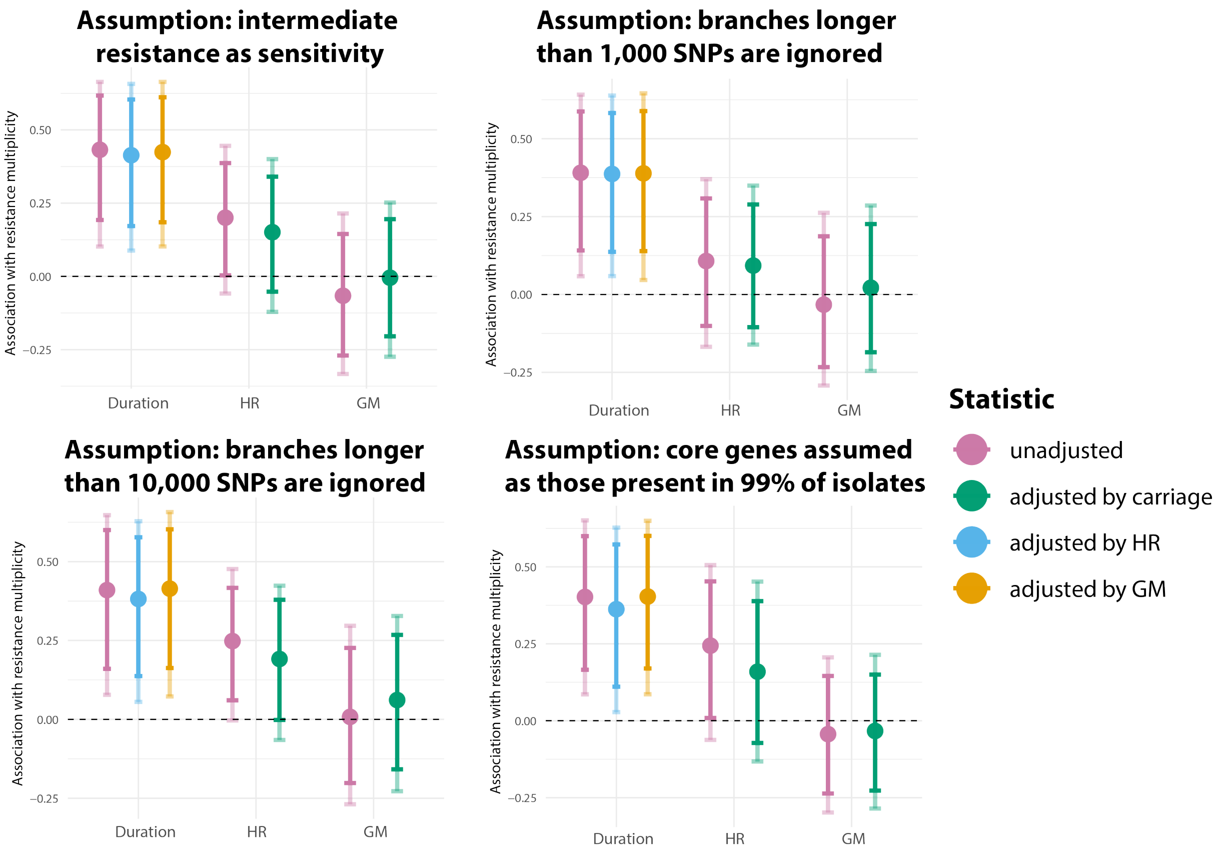

Figure S9. The impact of four assumptions on the main result in Figure 2: classification of intermediate resistance as sensitivity (as opposed to resistant in the main analysis) (top left), disregard of all branches longer than 1,000 SNPs (as opposed to 5,000 SNPs in the main analysis) to estimated HGT (top right), disregard of all branches longer than 10,000 SNPs to estimated HGT (bottom left), definition of the core genome as a concatenation of genes present in 99% of all isolates (as opposed to 70% in the main analysis) (bottom right).

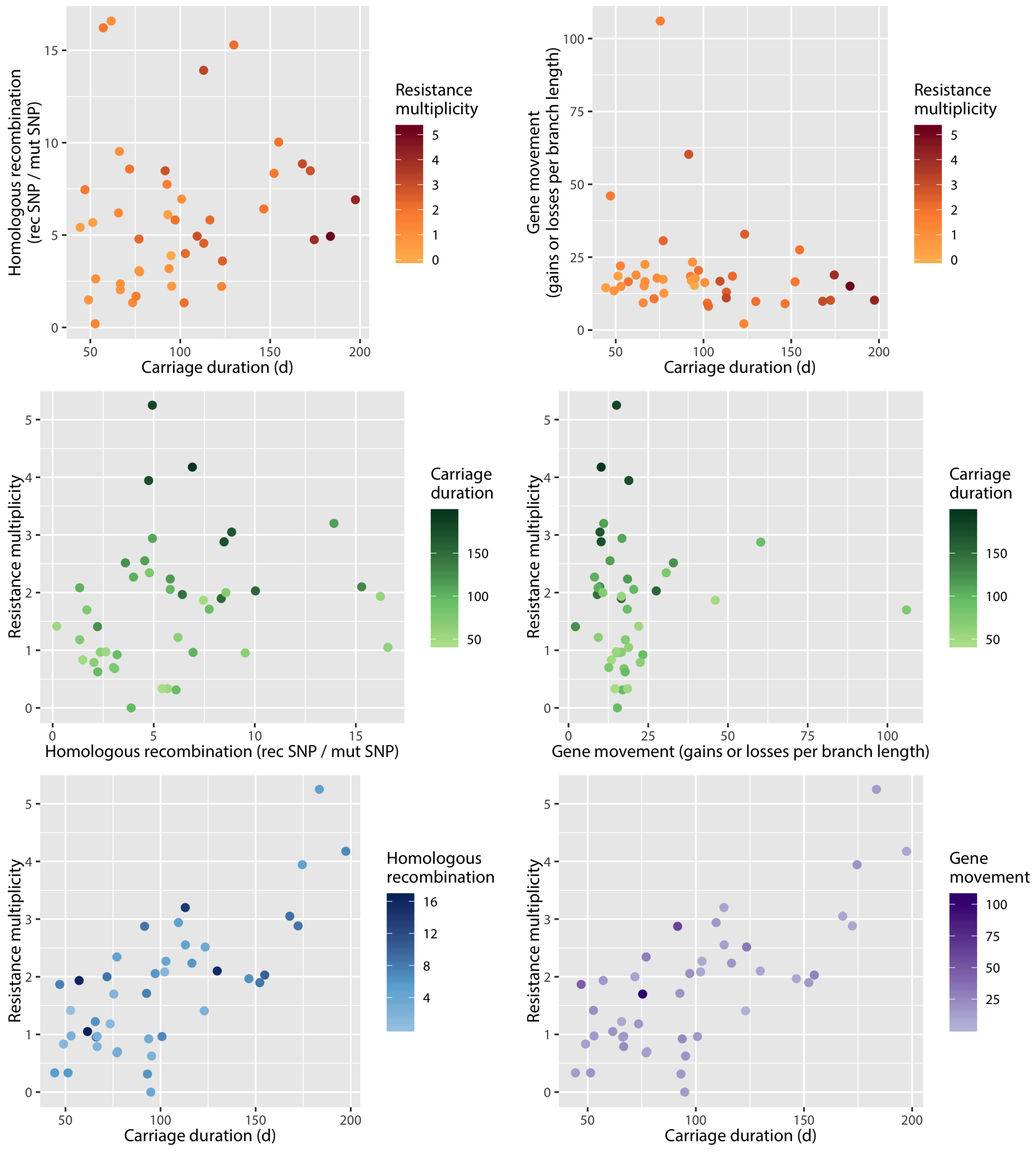

Figure S10. Scatter plots between the four main variables: duration of carriage, resistance multiplicity, homologous recombination rate and gene movement rate. Points are coloured according to the value of a variable that is not plotted. Each point corresponds to a per-cluster mean estimate.
